# 212. Measuring the Morbidity of Infectious Complications of the Opiate Epidemic: A Retrospective Cohort Study

**DOI:** 10.1093/ofid/ofab466.414

**Published:** 2021-12-04

**Authors:** Leah Harvey, Hassen Abdulkerim, Jacqueline Boudreau, Judith Strymish, Justeen Hyde, Allen Gifford, Westyn Branch-Elliman

**Affiliations:** 1 Boston Medical Center, Boston, Massachusetts; 2 Veterans Affairs Boston Center for Healthcare Organization and Implementation Research, Boston, Massachusetts; 3 VA Boston Healthcare System, West Roxbury, MA

## Abstract

**Background:**

Many states have reported that the incidence of invasive bacterial and viral infections has risen alongside rates of opiate use and injection drug use. The aim of this exploratory project is to characterize the incidence of invasive bacterial infections (IBI) over time in a national Veteran population, describe screening for substance use among Veterans with IBI, and assess engagement in harm reduction services.

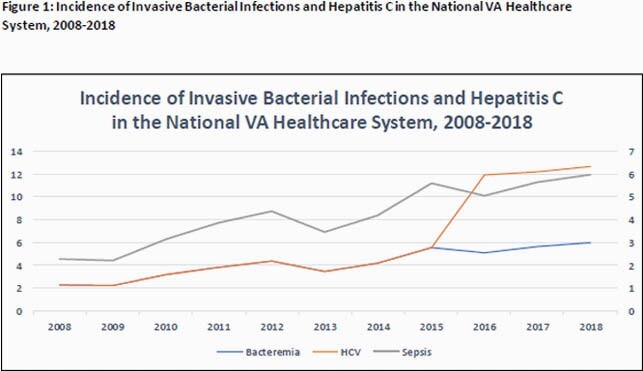

**Methods:**

A national, multicenter, retrospective cohort of Veterans admitted to the Veterans Health Administration (VA) between 10/1/2008 – 9/30/2018 with a positive blood culture was created using electronic health record (EHR) data. Patients’ demographics, clinical characteristics, microbiologic cultures, prescription history, laboratory values, and administrative coding data were extracted from the EHR. All analyses were performed in Microsoft Excel.

**Results:**

Among 5,158,137 inpatient admissions during the study period, we identified 257,926 unique patients with bacteremia (5.0%). The incidence of bacteremia/sepsis increased consistently during the study period, rising from 2.29 per 10,000 patient-days to 5.97 per 10,000 patient-days across the national VA healthcare system (Figure 1). Among Veterans with bacteremia, 17,436 (6.8%) had prior history of substance use and 24,927 (9.7%) had a history of hepatitis C virus infection. In 196,295 cases (76.1%), no urine toxicology screening was completed or the result was negative. 34,005 (13.2%) of patients with bacteremia had at least one urinary toxicology positive for opiates and of these, 6,173 (18.1%) had documentation of a prescription for either naltrexone or buprenorphine/naloxone prior to admission or on hospital discharge.

**Conclusion:**

Similar to findings in other populations, the incidence of IBI has steadily increased within the national VA. Despite limited screening, a high proportion of patients admitted to the VA with IBI were found to have underlying substance use. Additional work, including increased screening, is needed to assess the uptake of evidence-based interventions, such as naloxone, and to identify optimal strategies for improving adoption of other harm reduction services in this population.

**Disclosures:**

**All Authors**: No reported disclosures

